# Evaluating the Impact of a Good Clinical Practice Workshop on the Knowledge and Attitude of Postgraduate Medical Students

**DOI:** 10.7759/cureus.79674

**Published:** 2025-02-26

**Authors:** Ashwini Patel, Surendra K Padarya, Anjali Virani, Puja Singh

**Affiliations:** 1 Anaesthesiology, Chhindwara Institute of Medical Sciences, Chhindwara, IND; 2 Orthopaedics, Bundelkhand Medical College, Sagar, IND; 3 Ophthalmology, Bundelkhand Medical College, Sagar, IND; 4 Pathology, Jawaharlal Nehru Medical College, Wardha, IND; 5 Pathology, Bundelkhand Medical College, Sagar, IND

**Keywords:** attitude, clinical trials, gcp, good clinical practices, knowledge

## Abstract

Background

Good clinical practice (GCP) is critical for the safety of participants and the quality of data generated during clinical studies. A GCP workshop can enhance the knowledge and attitude of postgraduate medical students towards GCP. Thus, a study was needed to assess the impact of such a workshop. This study aims to assess the impact of such a workshop on GCP for postgraduate medical students.

Methodology

A GCP workshop was organized for postgraduate medical students. We evaluated the workshop's effectiveness using the Kirkpatrick model of training evaluation. We collected the participant's knowledge and attitude using pre- and post-tests, questionnaires, and their feedback. We applied the Shapiro-Wilk test and the Wilcoxon Signed Rank test to the collected data.

Results

A total of 79 postgraduate students from different academic years participated in the workshop. The Shapiro-Wilk test, z-test, and Wilcoxon Signed Rank test, with p-values < 0.05, clearly outlined the positive impact of the workshop on the participant’s knowledge and attitude. Box plots for pre- and post-test responses for knowledge and attitude reinforced this finding. The feedback identified the need for more interactive and case-based workshop delivery.

Conclusion

This study clearly outlines the positive impact of conducting a workshop on GCP for postgraduate medical students.

## Introduction

The Good Clinical Practice (GCP) framework is vital for ensuring the safety of participants and the quality of data generated during clinical studies. India’s National Medical Commission emphasizes that rigorous training in these practices is essential for the ethical conduct of clinical research [1]. Moreover, recent findings suggest that structured educational interventions significantly enhance the knowledge and attitudes of healthcare practitioners toward clinical trials [2].

Workshops specifically designed for postgraduate medical students aim to bridge the knowledge gap in GCP principles and foster positive attitudes toward clinical research [3]. Experts increasingly view integrating comprehensive training into medical curricula as necessary to develop competent researchers committed to ethical practices [4].

The evaluation of the impact of the GCP workshop is crucial in strengthening the foundational knowledge and attitudes of postgraduate medical students regarding clinical research. With the growing complexity of clinical trials and the increasing emphasis on ethical standards, well-informed future healthcare professionals play an essential role in upholding them.

This study assesses the effectiveness of such workshops by evaluating shifts in knowledge and attitude before and after participation. The insights garnered from this evaluation will contribute to refining educational strategies and improving the integration of GCP training within medical education programs, ultimately striving for higher standards in clinical research conduct.

## Materials and methods

This prospective study evaluated the effects of a GCP workshop on the knowledge and attitude of postgraduate medical students. A two-day workshop was hosted at Bundelkhand Medical College, Sagar, Madhya Pradesh, India, on September 11th and 12th, 2024. We collected pre-test and post-test data and feedback from the study participants to assess the impact on knowledge and attitude. The Kirkpatrick evaluation model's first two stages, reaction, and learning, were included in the study. All the study participants gave written informed consent. We depersonalized their data before using it further for research. The study was approved by the Institute Ethical Committee, Bundelkhand Medical College (approval number: IECBMC/DHR/2024/107).

Inclusion and exclusion criteria

All the postgraduate medical students of the medical college with no prior experience with a GCP workshop/training, who had enrolled for the workshop, and tendered written consent were included in this study. We excluded all postgraduate students who attended the workshop but refused to give their written consent and any participants with prior experience with GCP workshops.

Participant enrollment

We enrolled participants by disseminating information on departmental and residential hostel notice boards and WhatsApp (Meta Platforms, Inc., Menlo Park, California, United States) groups. Out of 92 workshop participants, five did not provide written consent, and eight had prior experience with a GCP workshop. Thus, effectively, 79 participants were assessed in the study.

Workshop details

A two-day workshop was conducted covering various aspects of good clinical practices. All the lectures were taken by senior faculty members from the academic cell and curriculum committee with relevant certifications and training. Day 1 included sessions: Introduction to GCP (1 hour), Ethical Principles and Regulatory Framework (1 hour), Roles and Responsibilities of Stakeholders (1.5 hours), Informed Consent Process (1.5 hours), and Clinical Trial Protocols and Documentation (1.5 hours). Sessions on Day 2 covered: Data Management and Safety Reporting (1.5 hours), Quality Assurance and Risk Management (1.5 hours), Monitoring and Site Management (1.5 hours), and GCP in Practice (2 hours).

Assessment of workshop impact

The effect on knowledge was assessed using the pre-test and post-test questionnaire (Appendix A), which consisted of 35 questions covering the following sections: Basics of GCP (5), Institutional Review Board (5), Participant Rights & Informed Consent (5), Study Design & Protocol Compliance (5), Safety Reporting & Adverse Events (5), Confidentiality & Data Integrity (5), and Regulatory Compliance (5). All the questions were multiple-choice questions (MCQs) with an equal weight of 1 mark each. 

The effect of the GCP workshop on the attitude of the participants was assessed with the help of a questionnaire with 20 questions covering sections (Appendix B): ethics (3), informed consent (3), compliance (3), data integrity (3), training & accountability (3), audits & quality assurance (3), and regulatory compliance (2). All the questions were measured using a five-point Likert scale (Strongly Disagree, Disagree, Neutral, Agree, Strongly Agree).

Participant’s feedback on the workshop was collected with the help of a questionnaire with 11 questions (Appendix C) measured on a five-point Likert scale (Strongly Disagree, Disagree, Neutral, Agree, Strongly Agree). The members of the medical education unit and academic cell evaluated the questionnaires for their face, content, criteria, and construct validity, ensuring they were relevant, clear, and suitable. We calculated Cronbach alphas to assess the reliability of the knowledge and attitude questionnaires.

Material

Presenters of the workshop used Microsoft PowerPoint (Microsoft Corporation, Redmond, Washington, United States) to present didactic lectures. The lectures addressed topics such as GCP, investigators' responsibilities, ethics committees, essential documentation, medical law, and audit, as well as international regulations and guidelines. We obtained all written consent from participants using paper documents. We used Google Forms (Google LLC, Mountain View, California, United States) to collect the pre-test and post-test results, which measured the impact on knowledge and attitude, as well as feedback.

Procedure

**Figure 1:**
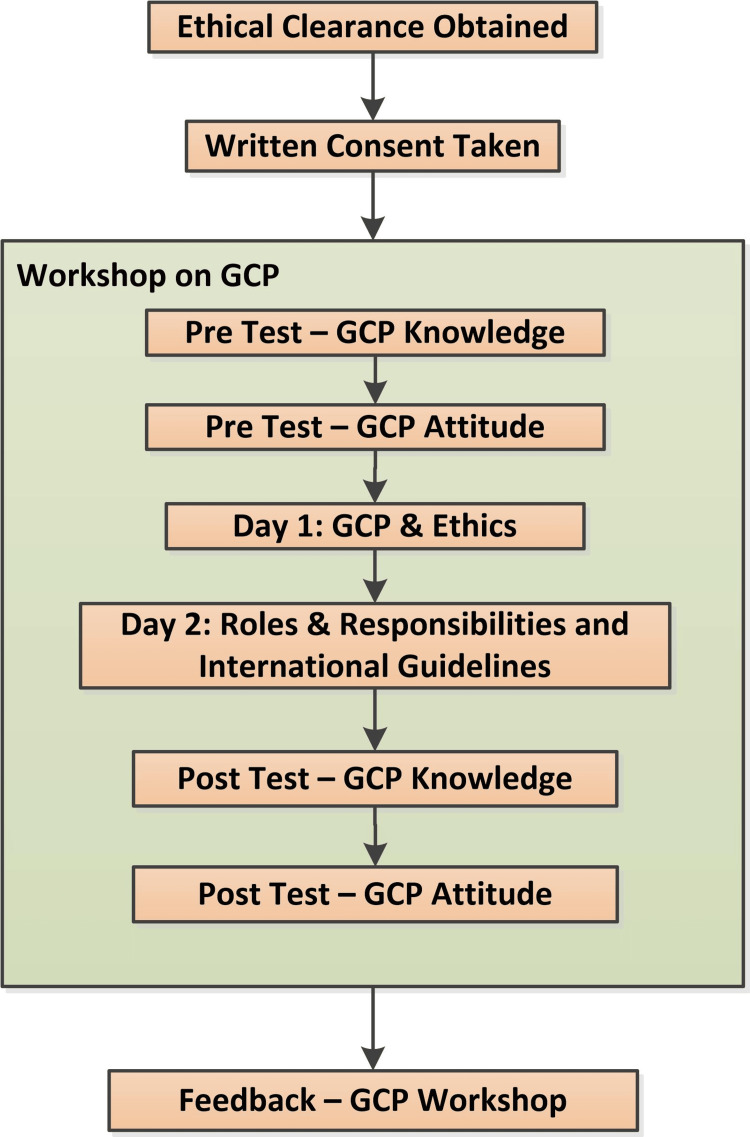
Schedule of events for the workshop on GCP GCP: Good Clinical Practice

Notice boards and various WhatsApp groups informed participants about the workshop. Upon registration, we requested participants to sign a written informed consent form. We conducted pre-tests to assess the participants' existing GCP knowledge and attitude. The workshop featured a variety of didactic lectures covering various topics related to GCP, ethics, etc. We conducted post-tests to gauge the shift in their GCP knowledge and attitude. We also collected feedback on the workshop. Figure 1 represents the schedule of the GCP workshop.

Statistical analysis

Z-scores were calculated for knowledge and attitude using pre-test and post-test data to evaluate the shift in the participants' central tendency. The pre-test and post-test distributions were established using the Shapiro test and evaluated the workshop effectiveness using a Wilcoxon signed-rank test. A p-value less than 0.05 was considered significant in the study. We performed the statistical analysis using Python (v3.9) (Python Software Foundation, Wilmington, Delaware, United States) and its libraries, namely Numpy (v1.24), Pandas (v1.3.5), Scipy (v1.14.1), and Seaborn (v0.11.2).

## Results

The demographic distribution of the study participants is presented in Table 1. The ratio of male to female participants was approximately 3:2. The majority of participants, comprising 60%, belonged to the 25-30 age group, with the remaining 40% being under 25 years old. The participants were evenly distributed among different years of postgraduate medical coursework. Most participants had no publication (81%), followed by one to two publications for 15%.

**Table 1:** Demographic distribution of the study participants (N=79)

Variable	Sub Class	Frequency	Percentage
Gender	Male	46	58.23
	Female	33	41.77
Age	< 25	31	39.24
	25-30	47	59.49
	> 30	1	1.27
Education	1^st^ Year	26	32.91
	2^nd^ Year	25	31.65
	3^rd^ year	28	35.44
Number of Publications	0 Paper	64	81.01
	1-2 Papers	12	15.19
	> 2 Papers	3	3.80

**Figure 2:**
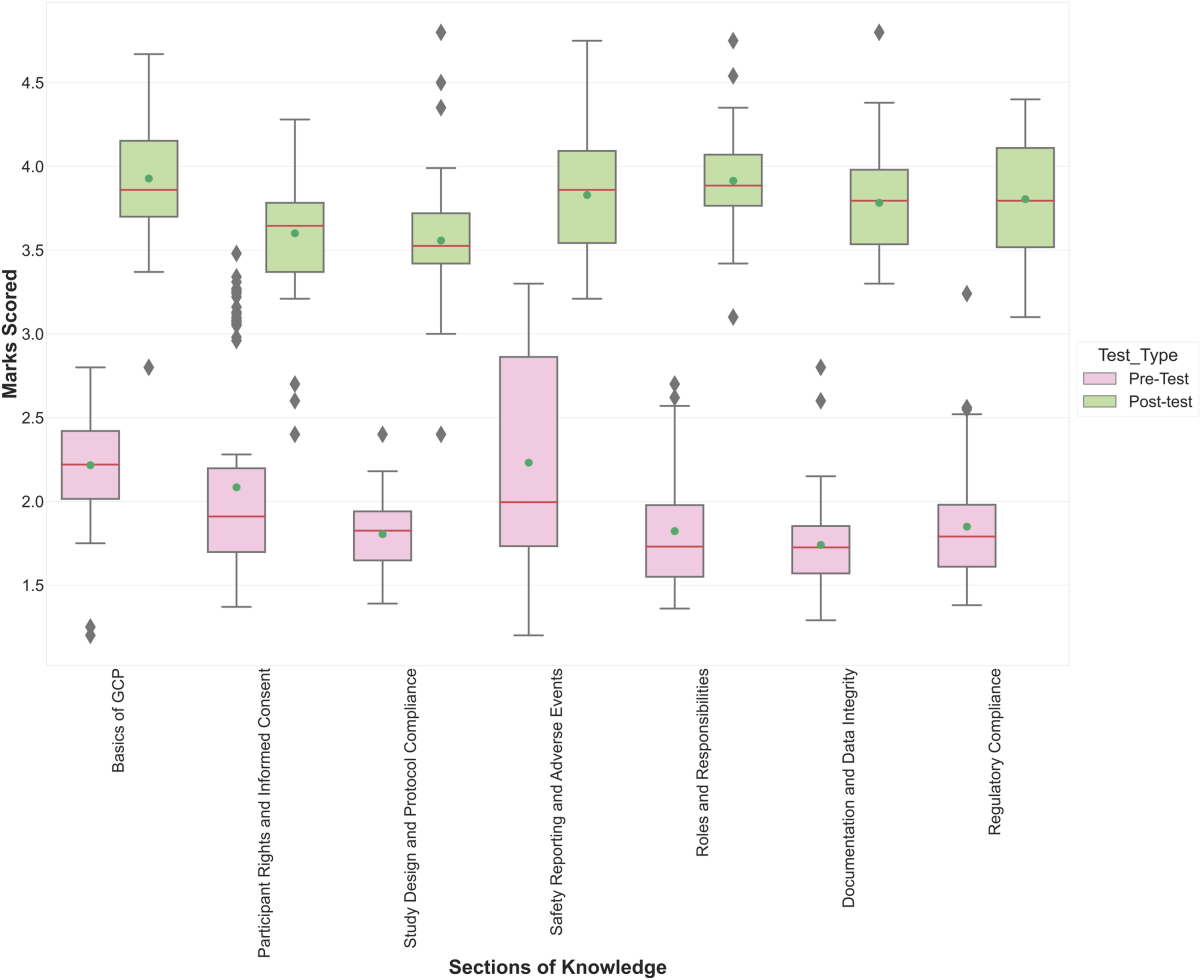
Distribution of participant’s scores in Pre-Test and Post-Test on different sections of knowledge of GCP GCP: Good Clinical Practice

Participant knowledge significantly improved after attending the GCP workshop. The overall average score increased from 1.93 ± 0.78 to 3.87 ± 0.65. The participants excelled in the knowledge sections on the basics of GCP, safety reporting and adverse events, and roles and responsibilities, achieving nearly four out of five correct responses (Figure 2).

**Figure 3:**
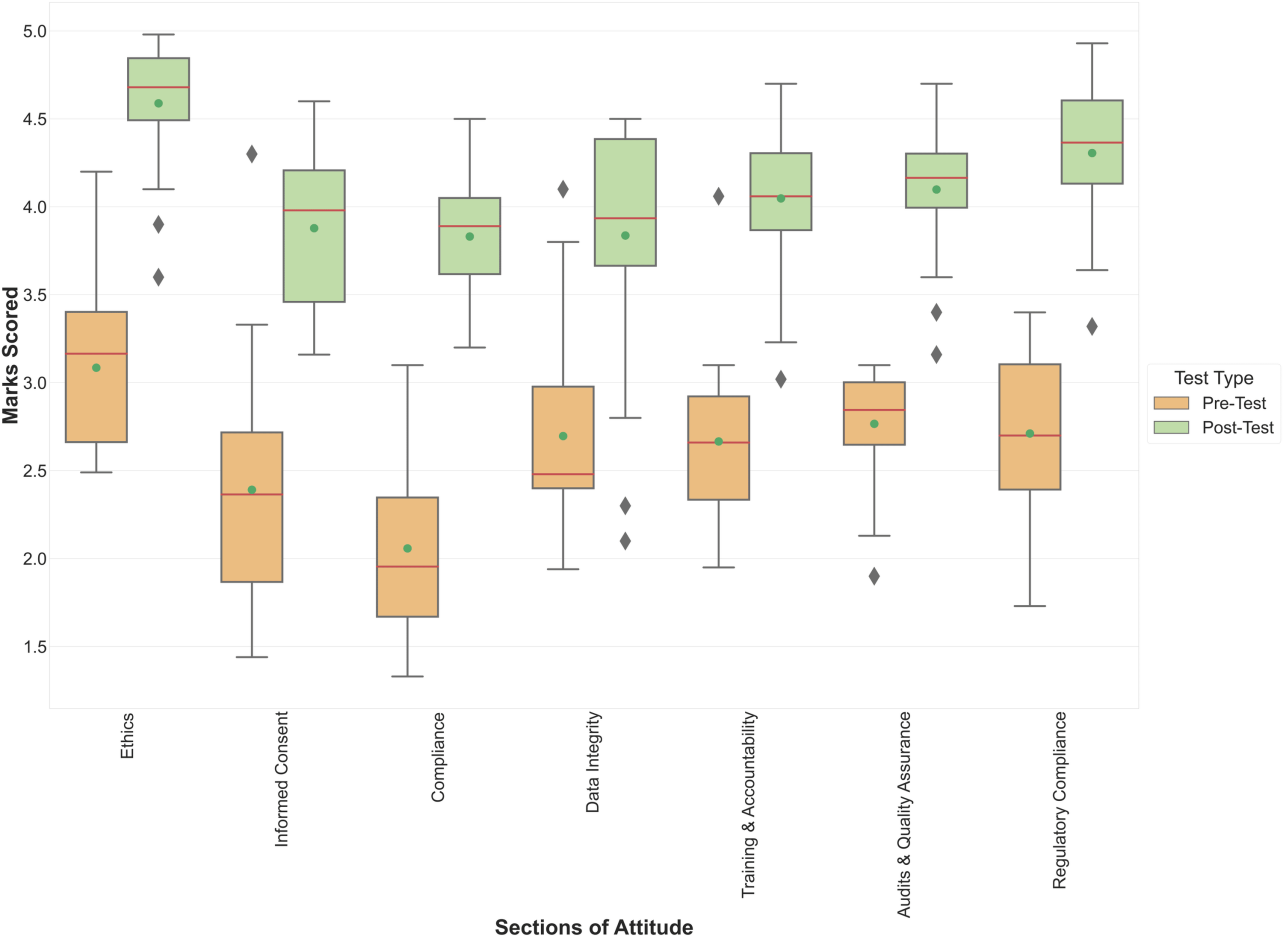
Distribution of participant’s scores in Pre-Test and Post-Test on different sections of attitude toward GCP GCP: Good Clinical Practice

Participant attitudes significantly improved after attending the GCP workshop. The overall average score increased from 2.61 ± 0.89 to 4.08 ± 0.75. Participants performed best in the ethics, audits and quality assurance, and regulatory compliance sections of attitude (Figure 3).

Table 2 presents the statistics to evaluate the GCP workshop's effectiveness in improving knowledge and attitude. Improvement in pre-test and post-test scores on questionnaires for knowledge and attitude outlines the workshop's effectiveness. We used the general Z-score test instead of the Mann-Whitney test for ordinal data because the sample size (n = 79) was significantly higher than 25. The Z-scores of knowledge and attitude were 85.14 and 22.19, respectively, with a p-value of ~0.0000. This indicated a significant increase in the post-test mean compared to the pre-test. To check if the pre- and post-test data was normally distributed or not, a Shapiro-Wilk test was performed. With very small p-values (< 0.05) for both the pre- and post-test, for both knowledge and attitude, it could be easily inferred that there was insufficient evidence that the distribution was normal. Statistical values of the Wilcoxon Signed Rank Test for knowledge and attitude were 0.00 and 47.00, respectively, with very small p-values (< 0.05). This outlined the effectiveness of the workshop on GCP. The Cronbach alphas for the knowledge and attitude assessment questionnaires were 0.761 and 0.727, respectively.

**Table 2:** Key statistics to assess the effectiveness of the workshop on GCP p-value  < 0.05 was considered as significant. GCP: Good Clinical Practice

Variable	Mean Distribution ( Pre vs Post)		Normal Distribution Check (Shapiro Test)				Wilcoxon Signed Rank Test	
			Pre Test Data		Post Test Data			
	Z-Score	p-Value	Statistic (W)	p-Value	Statistic (W)	p-Value	Statistic	p-Value
Knowledge	85.14	0	0.899	5.35E-21	0.99	0.000189	0.00	2.86E-116
Attitude	22.19	0	0.9858	0.000158	0.9583	0.000332	47.00	2.77E-24

Table 3 summarizes the participant's feedback on the GCP workshop. For all questions, except 5 and 8, more than 70% of the participants opted either for “Strongly Agreed” or “Agreed.” The Chi-Square test results showed that all the responses were statistically significant.

**Table 3:** Participant’s feedback on GCP workshop (N=79) Q1: The workshop content was relevant to my role and responsibilities. Q2: The objectives of the workshop were clearly defined and achieved. Q3: The facilitator(s) explained the concepts of Good Clinical Practice effectively. Q4: The interactive sessions (e.g., case studies, and group discussions) enhanced my learning. Q5: The materials provided (slides, handouts, etc.) were helpful and well-organized. Q6: The workshop improved my understanding of GCP principles and clinical trial regulations. Q7: I feel more confident in applying GCP principles in my research activities after attending the workshop. Q8: The workshop was well-structured and easy to follow. Q9: The time allocated for the workshop was sufficient for covering the topics. Q10: The venue/virtual platform and facilities were appropriate for learning. Q11: I would recommend this workshop to others involved in clinical research. χ2: Chi-Square Value. GCP: Good Clinical Practice

Question	Strongly Agree, n (%)	Agree, n (%)	Neutral, n (%)	Disagree, n (%)	Strongly Disagree, n (%)	χ2 Value	p-value
Q1	45 (56.96%)	29 (36.71%)	4 (5.06%)	1 (1.27%)	0 (0%)	103.47	0
Q2	26 (32.91%)	34 (43.04%)	11 (13.92%)	6 (7.59%)	2 (2.53%)	47.14	1.43E-09
Q3	38 (48.1%)	26 (32.91%)	10 (12.66%)	4 (5.06%)	1 (1.27%)	62.58	1.00E-12
Q4	30 (37.97%)	36 (45.57%)	8 (10.13%)	3 (3.8%)	2 (2.53%)	64.86	0
Q5	22 (27.85%)	33 (41.77%)	10 (12.66%)	12 (15.19%)	2 (2.53%)	36.25	2.57E-07
Q6	39 (49.37%)	24 (30.38%)	12 (15.19%)	4 (5.06%)	0 (0%)	63.85	0
Q7	33 (41.77%)	32 (40.51%)	10 (12.66%)	4 (5.06%)	0 (0%)	62.08	1.00E-12
Q8	24 (30.38%)	25 (31.65%)	18 (22.78%)	11 (13.92%)	1 (1.27%)	23.97	8.08E-05
Q9	35 (44.3%)	25 (31.65%)	15 (18.99%)	3 (3.8%)	1 (1.27%)	51.23	2.00E-10
Q10	25 (31.65%)	23 (29.11%)	22 (27.85%)	7 (8.86%)	2 (2.53%)	28.03	1.23E-05
Q11	42 (53.16%)	29 (36.71%)	5 (6.33%)	3 (3.8%)	0 (0%)	88.03	0

## Discussion

Guidelines for postgraduate medical education in India mandate a research project for the students. Srivastava et al. outlined the critical global role of GCP in preserving and protecting human rights [5]. Castelino et al. stated the lack of commitment of clinicians towards conducting proper clinical studies, quality control, and documentation, and humans being used as experimental replacements for animals are key concerns of GCP in India [6]. Thus, a proper awareness of the medical relevance of GCP is critical. A workshop on GCP can bridge the gap in research and ethics guidelines among the students. The objective of this study is to evaluate the effect of conducting a workshop on GCP on knowledge and attitude among postgraduate medical students.

Postgraduate medical students participated in this study. Various researchers experimented with different compositions for their study groups. Harshita et al. included a wide variety of medical researchers as participants [3]. Azakir et al. included physicians in their study [7]. Than et al. covered postgraduate medical students [8]. The composition of the study group, age group, and prior research experience varied from study to study.

Figure 2 outlines that all postgraduate students have a basic awareness of ethics and good clinical practices and their importance. Many researchers have assessed the participants' level of existing knowledge in their studies. Than et al. [8], Al Demour et al. [9], Azakir et al. [7], and Choudhury et al. [10] identified that researchers have limited knowledge about research ethics, ethical committees, and their importance in their research.

Figure 2 also shows that the workshop significantly improved (by close to 40%) the participants' knowledge in the areas of documentation and data integrity, regulatory compliance, and roles and responsibilities. Awatagiri et al. in their study noticed that, as a result of a planned training session, the participant’s knowledge increased in all areas except about ethical committees [11]. SureshBabu et al. [4] and Goel et al. [2] also noticed a significant improvement in knowledge due to such educational intervention. Vora et al. also saw improvements in knowledge ranging from 30% to 70% in response to certain questions [12]. Yeoh et al. conducted a study on the benefits of conducting GCP training 22 times since 2016 [13]. They noticed an increase in knowledge by about 20% on average. Felaefel et al. in their study identified that lack of ethics training was a significant predictor of scientific misconduct [14]. Therefore, such workshops can significantly enhance the participant's understanding.

Mozersky et al. conducted a mixed-methods study [15]. They emphasized that in addition to formal training, an individual's experience, practice, and observation of peers and seniors play a critical role in improving knowledge. 

Figure 3 demonstrates a marginal improvement in the participants' attitudes compared to their prior knowledge. Than et al. observed that participants' attitudes were suboptimal in areas of informed consent, data fabrication, and ethical review board approval [8]. Al Demour et al. observed that participants' attitudes were subpar in matters related to patient consent and the ethical review committee [9]. In this study, participants' attitudes significantly improved after attending the workshop. Other studies have not measured the effect of workshops on participants' attitudes.

There are many training evaluation models; however, the Kirkpatrick Program evaluation model stands out among them. The study design evaluates the first two stages of the Kirkpatrick Program evaluation Model, namely reaction and learning. This study evaluated the impact of training and workshops on participants' knowledge and attitudes. Awatagiri et al. also used this evaluation model to assess the workshop's effectiveness on GCP [11]. Singh et al. used this model to evaluate the effectiveness of a portfolio workshop [16]. A long-term study is required to assess the long-term effect of such a workshop on the later two stages of the Kirkpatrick Program Evaluation Model, namely behavior and results. 

SureshBabu et al. [4] and Yeoh et al. [13] used a paired t-test for knowledge. This study used a z-test to compare the pre- and post-data means. Additionally, this study measured its effectiveness using the Wilcoxon signed rank test. Awatagiri et al. used the same approach [11]. Goel et al. used a two-tailed z-test to assess the impact on knowledge [2]. The Wilcoxon Signed Rank Test, a non-parametric test, doesn't require assumptions about the data distribution and is used to compare the medians of two related groups, making it a suitable alternative to the paired T-test when the data is not normally distributed. This study used the Wilcoxon signed rank test while other studies either t-test or z-test. This makes the findings of this study more significant.

Upon evaluating the feedback of the participants, it is evident that participants expect improvement in the training materials and delivery of the content. Thus, including case studies-based learning and making lectures more interactive will further improve both the knowledge and attitude of the participants.

Limitations

A long-term study will assist in precisely measuring the effects of GCP workshops on medical students and faculty at two additional levels, namely behaviors and results. This study does not include Bachelor of Medicine, Bachelor of Surgery (MBBS) (graduate medical students) and faculty as participants. This is the largest community of researchers in the college. Thus, including them in the study would have increased the effectiveness of its findings. Evaluating the impact of such a workshop on the practice of GCP can bring out the practical aspects and challenges faced by researchers in incorporating GCP in their research work. Additionally, questionnaires with higher reliability can increase the reliability of the study.

## Conclusions

GCP plays a pivotal role in clinical trials to ensure the study participants’ well-being and the significance and validity of the research findings. The findings of this study clearly outline the positive impact of conducting a workshop on GCP on the knowledge and attitude of postgraduate medical students. Such a workshop will not only improve the awareness and knowledge of participants but also improve their attitude. However, it will be worth evaluating the long-term impact of such a workshop on the practices, attitudes, and knowledge of the participants. In addition, enhancing the training material and delivery method can significantly improve the workshop's effectiveness.

## Appendices

Appendix A: Impact on knowledge evaluation: pre-test and post-test questionnaire

Section 1: Basics of GCP

1. What is the primary goal of Good Clinical Practice (GCP)? a) Ensure data is published quickly b) Ensure participants' safety and data reliability c) Reduce the cost of clinical trials d) Promote pharmaceutical marketing

2. Which document outlines ethical principles for human research? a) ICH-GCP Guidelines b) Declaration of Helsinki c) Nuremberg Code d) Belmont Report

3. Which of the following best defines a protocol? a) Financial agreement between the sponsor and investigator b) A document that describes the objectives, design, and methodology of the trial c) A schedule for clinical site visits d) An informed consent form template

4. Who is primarily responsible for the conduct of the trial at the site? a) Sponsor b) Principal Investigator (PI) c) Ethics Committee d) Clinical Research Organization

5. What is the purpose of the Ethics Committee (EC)? a) Approve drug marketing applications b) Ensure the trial follows GCP guidelines c) Ensure the rights, safety, and well-being of participants d) Monitor sponsor performance

Section 2: Participant Rights and Informed Consent

6. When should informed consent be obtained from a participant? a) Before the trial starts b) After the first dose of the trial medication c) Only if the participant asks d) At the end of the trial

7. What should be included in the informed consent form? a) Participant’s employment history b) Compensation details, risks, and expected benefits c) Drug manufacturing details d) Sponsor’s financial statements

8. Can a participant withdraw from the study at any time? a) Yes, without any penalty b) Only after completing half of the study c) Only if they pay compensation d) No, once enrolled, they must complete the study

9. How should vulnerable populations (e.g., children or unconscious patients) be included in a study? a) With additional safeguards and, where applicable, consent from guardians b) As long as they are available for the study c) Only if no other participants are available d) With the sponsor’s approval only

10. What should the investigator do if new information arises that may affect a participant's willingness to continue? a) Do nothing if the trial is already ongoing b) Update the informed consent and discuss it with the participant c) Report to the sponsor only d) Wait until the trial concludes

Section 3: Study Design and Protocol Compliance

11. What is a protocol deviation? a) A significant modification to the trial's objectives b) A change in the study plan without prior approval c) A mistake in data entry d) A minor typographical error in the protocol

12. When can the protocol be amended? a) Only with approval from the sponsor and Ethics Committee b) At the investigator’s discretion c) After recruiting all participants d) Whenever a participant requests a change

13. Which phase of clinical trials focuses on assessing drug safety? a) Phase I b) Phase II c) Phase III d) Phase IV

14. What is the role of the Data Safety Monitoring Board (DSMB)? a) Provide funding for trials b) Ensure regulatory compliance c) Monitor participant safety and review trial data periodically d) Promote the trial in public forums

15. What is randomization in clinical trials? a) Selecting participants based on convenience b) Assigning participants randomly to treatment groups c) Using the same treatment for all participants d) Choosing participants who meet the protocol criteria

Section 4: Safety Reporting and Adverse Events

16. What is an adverse event (AE)? a) Any positive outcome from the trial b) Any undesirable medical occurrence in a participant, regardless of its relationship to the treatment c) A protocol violation d) An incident that results in trial termination

17. What is a serious adverse event (SAE)? a) An event leading to participant death or hospitalization b) A minor headache reported by a participant c) A delay in data submission d) An event that does not require immediate attention

18. Within how many days should an SAE be reported to the Ethics Committee? a) 30 days b) 7 days c) 24 hours d) 10 days

19. Who is responsible for reporting adverse events during a clinical trial? a) Principal Investigator b) Sponsor c) Ethics Committee d) Data Monitoring Board

20. How should unexpected adverse events be handled? a) Stop the trial immediately b) Document, report to relevant authorities, and evaluate the event’s impact c) Ignore if they are unrelated to the study drug d) Inform participants only at the end of the study

Section 5: Roles and Responsibilities

21. Who is responsible for monitoring the trial to ensure compliance with GCP? a) Ethics Committee b) Sponsor or Contract Research Organization (CRO) c) Investigator d) Government agencies

22. What is the role of the Sponsor? a) Provide drug samples only b) Fund the trial and oversee trial conduct c) Approve informed consent forms d) Conduct trial site visits

23. What must be documented in the Trial Master File (TMF)? a) Only financial reports b) All essential documents related to trial management c) Sponsor’s marketing plans d) Recruitment advertisements

24. How long must clinical trial data be retained? a) 1 year b) 5 years c) 15 years or as per local regulations d) Until the drug is marketed

25. When should the final report of a trial be submitted to regulatory authorities? a) As soon as the trial begins b) Only after participant follow-up is complete c) Within 3 months of trial completion d) After analyzing all the collected data

Section 6: Documentation and Data Integrity

26. What is source data verification (SDV)? a) Reviewing raw data from external sources b) Cross-checking trial data with original medical records c) Monitoring for regulatory compliance d) Checking financial statements

27. What should be done if a participant loses their informed consent form? a) Issue a duplicate form immediately b) Ask the Ethics Committee for permission to continue c) Re-consent the participant and document the process d) Remove the participant from the trial

28. What is the primary principle of data management in GCP? a) Collect as much data as possible b) Ensure data integrity, accuracy, and confidentiality c) Use only electronic data systems d) Minimize data collection to save costs

29. What should the investigator do if they detect fraudulent data entry? a) Report it to the sponsor and Ethics Committee b) Correct the data silently c) Inform participants d) Ignore it if the trial is almost complete

30. What is the preferred method for recording data during a trial? a) On sticky notes for convenience b) In a validated Case Report Form (CRF) c) On personal notes of the investigator d) In any digital format

Section 7: Regulatory Compliance

31. Which organization regulates clinical trials in India? a) CDSCO b) FDA c) WHO d) ICMR

32. What must be done if a trial drug is accidentally administered to the wrong participant? a) Report as a protocol deviation and follow reporting procedures b) Ignore if no harm occurs c) Withdraw the participant immediately d) Inform the sponsor only

33. Which phase of clinical trials monitors the long-term effects of a treatment? a) Phase I b) Phase II c) Phase III d) Phase IV

34. What is the ICH in the context of GCP? a) International Council for Harmonisation b) Institute of Clinical Health c) International Collaboration Hub d) Indian Council of Health

35. What should the investigator do if a participant wishes to see their trial data? a) Provide access following ethical guidelines b) Deny access to maintain confidentiality c) Refer the participant to the sponsor d) Only provide data after trial completion

Appendix B: Impact on attitude evaluation: pre-test and post-test questionnaire

(Likert Scale: Strongly Disagree, Disagree, Neutral, Agree, Strongly Agree)

Ethics

1.       The primary goal of GCP is to protect the rights and well-being of participants in clinical research..

2.       Ethical guidelines under GCP ensure that participants are not exposed to unnecessary risks.

3.       GCP helps foster trust between researchers and participants by prioritizing participant welfare.

Informed Consent

4.       The informed consent process is essential for maintaining transparency in clinical trials.

5.       Participants should have the right to withdraw from a trial at any point without penalty or pressure.

6.       The language used in informed consent forms should be simple and easy for participants to understand.

Compliance

7.       Adhering to the study protocol is critical to achieving reliable and reproducible research results.

8.       Deviating from approved clinical protocols compromises the scientific validity of the trial.

9.       I believe that strict compliance with GCP may sometimes hinder the flexibility required in real-world clinical research.

Data Integrity

10.   Maintaining accurate and complete documentation is a crucial aspect of GCP compliance.

11.   Ensuring the quality and integrity of data is essential for obtaining credible research outcomes.

12.   Data falsification or fabrication, even on a small scale, undermines the entire research process.

Training & Accountability

13.   Regular GCP training should be mandatory for all members of the research team.

14.   Adequate awareness of GCP guidelines improves the efficiency of clinical trial conduct.

15.   Every member of a research team should understand their individual responsibilities under GCP.

Audits & Quality Assurance

16.   Monitoring and auditing are essential for ensuring compliance with GCP standards.

17.   I believe audits help identify areas of improvement in clinical trials, making them more effective.

18.   The presence of external monitors increases transparency and accountability in clinical research.

Regulatory Compliance

19.   GCP compliance is necessary to meet both national and international regulatory requirements.

20.   Despite the challenges, following GCP guidelines is essential to conducting ethical and high-quality clinical research.

Appendix C: Feedback questionnaire - GCP workshop 

(Likert Scale: Strongly Disagree, Disagree, Neutral, Agree, Strongly Agree) 1. The workshop content was relevant to my role and responsibilities. 2. The objectives of the workshop were clearly defined and achieved. 3. The facilitator(s) explained the concepts of Good Clinical Practice effectively. 4. The interactive sessions (e.g., case studies, and group discussions) enhanced my learning. 5. The materials provided (slides, handouts, etc.) were helpful and well-organized. 6. The workshop improved my understanding of GCP principles and clinical trial regulations. 7. I feel more confident in applying GCP principles in my research activities after attending the workshop. 8. The workshop was well-structured and easy to follow. 9. The time allocated for the workshop was sufficient for covering the topics. 10. The venue/virtual platform and facilities were appropriate for learning. 11. I would recommend this workshop to others involved in clinical research.
 
